# 1-[Bis(4-fluoro­phen­yl)meth­yl]piperazine

**DOI:** 10.1107/S1600536812036902

**Published:** 2012-08-31

**Authors:** A. S. Dayananda, Grzegorz Dutkiewicz, H. S. Yathirajan, A. R. Ramesha, Maciej Kubicki

**Affiliations:** a Department of Studies in Chemistry, University of Mysore, Mysore 570 006, India; b Department of Chemistry, Adam Mickiewicz University, Grunwaldzka 6, 60-780 Poznań, Poland; c R. L. Fine Chem., Bengaluru 560 064, India

## Abstract

In the title mol­ecule, C_17_H_18_F_2_N_2_, the dihedral angle between the benzene rings is 73.40 (3)°. The piperazine ring is close to an ideal chair conformation and the N—H hydrogen is in an equatorial position. In the crystal, molecules are linked *via* weak C—H⋯F hydrogen bonds.

## Related literature

For medical applications of piperazines, see: Bogatcheva *et al.* (2006 ▶); Brockunier *et al.* (2004 ▶). For related structures, see: Betz *et al.* (2011*a* ▶,*b* ▶); Hu *et al.* (2003 ▶); Naveen *et al.* (2006 ▶). For asymmetry parameters, see: Duax & Norton (1975 ▶).
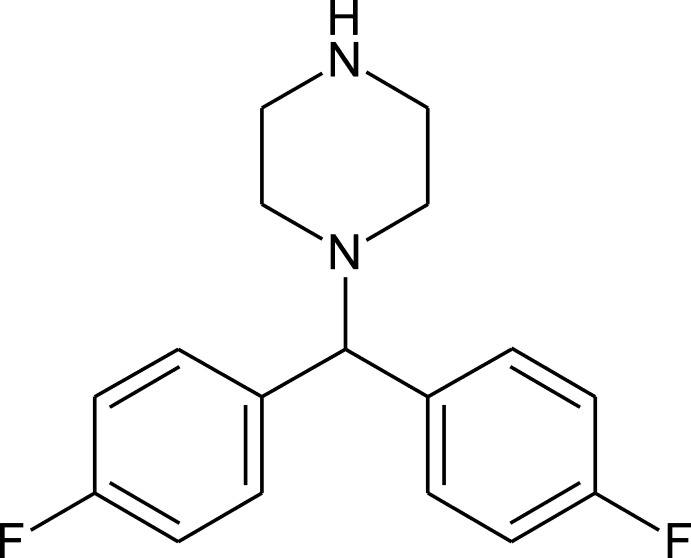


## Experimental

### 

#### Crystal data

C_17_H_18_F_2_N_2_
*M* *_r_* = 288.33
Monoclinic, 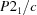

*a* = 12.1574 (5) Å
*b* = 8.8559 (2) Å
*c* = 13.8604 (4) Å
β = 93.355 (3)°
*V* = 1489.72 (8) Å^3^
*Z* = 4
Cu *K*α radiation
μ = 0.77 mm^−1^
*T* = 130 K
0.15 × 0.08 × 0.06 mm

#### Data collection

Atlas SuperNova (Single source at offset) diffractometer
Absorption correction: multi-scan (*CrysAlis PRO*; Agilent, 2011 ▶) *T*_min_ = 0.828, *T*_max_ = 1.000
8816 measured reflections
3006 independent reflections
2847 reflections with *I* > 2σ(*I*)
*R*_int_ = 0.010

#### Refinement

*R*[*F* ^2^ > 2σ(*F* ^2^)] = 0.035
*wR*(*F* ^2^) = 0.096
*S* = 1.04
3006 reflections
262 parameters
All H-atom parameters refined
Δρ_max_ = 0.18 e Å^−3^
Δρ_min_ = −0.23 e Å^−3^

### 

Data collection: *CrysAlis PRO* (Agilent, 2011 ▶); cell refinement: *CrysAlis PRO*; data reduction: *CrysAlis PRO*; program(s) used to solve structure: *SIR92* (Altomare *et al.*, 1993 ▶); program(s) used to refine structure: *SHELXL97* (Sheldrick, 2008 ▶); molecular graphics: *XP* in *SHELXTL* (Sheldrick, 2008 ▶); software used to prepare material for publication: *SHELXL97*.

## Supplementary Material

Crystal structure: contains datablock(s) I, global. DOI: 10.1107/S1600536812036902/mw2085sup1.cif

Structure factors: contains datablock(s) I. DOI: 10.1107/S1600536812036902/mw2085Isup2.hkl

Supplementary material file. DOI: 10.1107/S1600536812036902/mw2085Isup3.cml

Additional supplementary materials:  crystallographic information; 3D view; checkCIF report

## Figures and Tables

**Table 1:** Hydrogen-bond geometry (Å, °)

*D*—H⋯*A*	*D*—H	H⋯*A*	*D*⋯*A*	*D*—H⋯*A*
C25—H25⋯F24^i^	0.962 (14)	2.424 (14)	3.2720 (13)	146.8 (10)
C25—H25⋯F34^ii^	0.962 (14)	2.533 (14)	3.1998 (13)	126.5 (10)

Symmetry codes: (i) 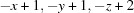; (ii) 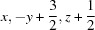.

**Fig. 1.**
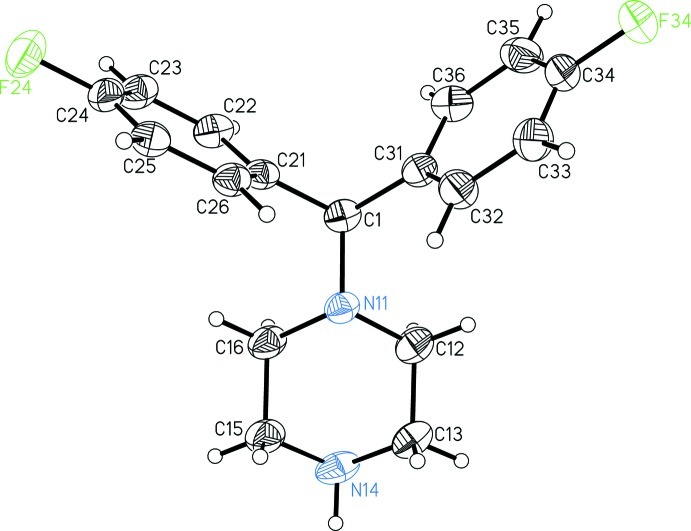
Perspective view of I together with the atom labelling scheme. The ellipsoids are drawn at the 50% probability level and H-atoms are depicted as spheres with arbitrary radii.

## supplementary crystallographic information

### Comment

Piperazine is currently the most important building block used in drug discovery with a high number of positive hits encountered in biological screens of this heterocycle and its congeners. They are found in biologically active compounds across a number of different therapeutic areas such as antifungal, antibacterial, antimalarial, antipsychotic, antidepressant and antitumour activity against colon, prostate, breast, lung and leukemia tumors (Brockunier *et al.*, 2004; Bogatcheva *et al.*, 2006). 1-[bis(4-fluorophenyl)methyl]piperazine is an intermediate for the preparation of flunarizine which is a calcium channel blocker. Here we report the crystal structure of the title compound (I).

Only two structures of neutral 1-benzhydrylpiperazine derivatives have been reported so far: 1-benzhydrylpiperazine itself (Naveen *et al.*, 2006) and (*R*)-1-((4-chlorophenyl)phenylmethyl)piperazine (Hu *et al.*, 2003) as well as two structures of salts of I, the trinitrophenolate (Betz *et al.*, 2011*a*) and the 2-(2-phenylethyl)benzoate (Betz *et al.*, 2011*b*).

The dihedral angle between the mean planes of the *p*-fluorophenyl rings is 73.40 (3)°. The piperazine ring is in a chair conformation and the asymmetry parameters (Duax & Norton, 1975) are quite small with the largest value for the mirror plane being 3.7° and for the twofold axis 3.0°. The N—H hydrogen atom is in an equatorial position (the C—C—N—H torsion angles are 177° and -176°). In the crystal there are only very weak C—H···F contacts and, interestingly, the shortest contacts to both F atoms are created by the same carbon atom (C25). Therefore it seems that the three-dimensional structure is mainly governed by van der Waals forces.

### Experimental

The title compound obtained as a gift sample from *R. L*. Fine Chem., Bengaluru, India. X-ray quality crystals were grown from a 1:1 (v:v) toluene/hexane solution by slow evaporation (m.p: 360–362 K).

### Refinement

The hydrogen atoms were freely refined.

### Crystal data

**Table:**

C_17_H_18_F_2_N_2_	*F*(000) = 608
*M**_r_* = 288.33	*D*_x_ = 1.286 Mg m^−^^3^
Monoclinic, *P*2_1_/*c*	Cu *K*α radiation, λ = 1.5418 Å
Hall symbol: -P 2ybc	Cell parameters from 110 reflections
*a* = 12.1574 (5) Å	θ = 2.9–27.8°
*b* = 8.8559 (2) Å	µ = 0.77 mm^−^^1^
*c* = 13.8604 (4) Å	*T* = 130 K
β = 93.355 (3)°	Block, colourless
*V* = 1489.72 (8) Å^3^	0.15 × 0.08 × 0.06 mm
*Z* = 4	

### Data collection

**Table:**

Atlas SuperNova (Single source at offset) diffractometer	3006 independent reflections
Radiation source: SuperNova (Cu) X-ray Source	2847 reflections with *I* > 2σ(*I*)
Mirror monochromator	*R*_int_ = 0.010
Detector resolution: 10.5357 pixels mm^-1^	θ_max_ = 75.3°, θ_min_ = 3.6°
ω scan	*h* = −15→15
Absorption correction: multi-scan (*CrysAlis PRO*; Agilent, 2011)	*k* = −9→10
*T*_min_ = 0.828, *T*_max_ = 1.000	*l* = −14→17
8816 measured reflections	

### Refinement

**Table:**

Refinement on *F*^2^	Primary atom site location: structure-invariant direct methods
Least-squares matrix: full	Secondary atom site location: difference Fourier map
*R*[*F*^2^ > 2σ(*F*^2^)] = 0.035	Hydrogen site location: difference Fourier map
*wR*(*F*^2^) = 0.096	All H-atom parameters refined
*S* = 1.04	*w* = 1/[σ^2^(*F*_o_^2^) + (0.0518*P*)^2^ + 0.3446*P*] where *P* = (*F*_o_^2^ + 2*F*_c_^2^)/3
3006 reflections	(Δ/σ)_max_ = 0.001
262 parameters	Δρ_max_ = 0.18 e Å^−^^3^
0 restraints	Δρ_min_ = −0.23 e Å^−^^3^

### Special details

**Table:**

Geometry. All s.u.'s (except the s.u. in the dihedral angle between two l.s. planes) are estimated using the full covariance matrix. The cell s.u.'s are taken into account individually in the estimation of s.u.'s in distances, angles and torsion angles; correlations between s.u.'s in cell parameters are only used when they are defined by crystal symmetry. An approximate (isotropic) treatment of cell s.u.'s is used for estimating s.u.'s involving l.s. planes.
Refinement. Refinement of *F*^2^ against ALL reflections. The weighted *R*-factor *wR* and goodness of fit *S* are based on *F*^2^, conventional *R*-factors *R* are based on *F*, with *F* set to zero for negative *F*^2^. The threshold expression of *F*^2^ > 2σ(*F*^2^) is used only for calculating *R*-factors(gt) *etc*. and is not relevant to the choice of reflections for refinement. *R*-factors based on *F*^2^ are statistically about twice as large as those based on *F*, and *R*- factors based on ALL data will be even larger.

### Fractional atomic coordinates and isotropic or equivalent isotropic displacement parameters (Å^2^)

**Table:**

	*x*	*y*	*z*	*U*_iso_*/*U*_eq_	
C1	0.71855 (8)	0.26307 (12)	0.64093 (7)	0.0297 (2)	
H1	0.6808 (10)	0.1909 (14)	0.5950 (9)	0.031 (3)*	
N11	0.82339 (7)	0.19519 (10)	0.67957 (6)	0.0296 (2)	
C12	0.89231 (9)	0.15265 (14)	0.59996 (9)	0.0377 (3)	
H12B	0.9123 (11)	0.2450 (16)	0.5634 (10)	0.040 (3)*	
H12A	0.8512 (12)	0.0821 (16)	0.5550 (10)	0.044 (4)*	
C13	0.99715 (9)	0.07353 (14)	0.63834 (9)	0.0388 (3)	
H13B	1.0416 (12)	0.0469 (16)	0.5818 (11)	0.048 (4)*	
H13A	1.0429 (11)	0.1472 (16)	0.6811 (10)	0.041 (3)*	
N14	0.96894 (8)	−0.06217 (11)	0.69096 (8)	0.0400 (2)	
H14	1.0301 (12)	−0.1145 (17)	0.7121 (10)	0.047 (4)*	
C15	0.90455 (10)	−0.01932 (14)	0.77224 (10)	0.0428 (3)	
H15B	0.9468 (12)	0.0538 (18)	0.8199 (10)	0.050 (4)*	
H15A	0.8848 (12)	−0.1141 (18)	0.8085 (11)	0.054 (4)*	
C16	0.79931 (9)	0.05778 (13)	0.73443 (10)	0.0388 (3)	
H16B	0.7563 (12)	−0.0116 (17)	0.6926 (10)	0.046 (4)*	
H16A	0.7553 (11)	0.0865 (16)	0.7897 (10)	0.043 (4)*	
C21	0.64243 (8)	0.29345 (11)	0.72207 (7)	0.0282 (2)	
C22	0.53738 (9)	0.23052 (12)	0.71842 (9)	0.0343 (2)	
H22	0.5114 (11)	0.1663 (16)	0.6633 (10)	0.040 (3)*	
C23	0.46608 (9)	0.25595 (13)	0.79170 (9)	0.0379 (3)	
H23	0.3926 (13)	0.2108 (17)	0.7896 (11)	0.051 (4)*	
C24	0.50228 (9)	0.34553 (12)	0.86750 (8)	0.0344 (2)	
F24	0.43371 (6)	0.37247 (9)	0.93966 (5)	0.0496 (2)	
C25	0.60588 (9)	0.41045 (12)	0.87452 (8)	0.0324 (2)	
H25	0.6270 (11)	0.4725 (16)	0.9295 (10)	0.038 (3)*	
C26	0.67547 (8)	0.38383 (12)	0.80075 (8)	0.0299 (2)	
H26	0.7491 (12)	0.4266 (15)	0.8047 (10)	0.042 (3)*	
C31	0.73448 (8)	0.40781 (12)	0.58434 (7)	0.0301 (2)	
C32	0.81328 (9)	0.51586 (13)	0.61203 (8)	0.0369 (3)	
H32	0.8644 (12)	0.4954 (16)	0.6675 (10)	0.045 (4)*	
C33	0.82205 (10)	0.65060 (14)	0.56149 (9)	0.0418 (3)	
H33	0.8754 (13)	0.7275 (19)	0.5793 (11)	0.056 (4)*	
C34	0.75027 (10)	0.67407 (13)	0.48296 (9)	0.0401 (3)	
F34	0.75839 (7)	0.80528 (9)	0.43211 (6)	0.0570 (2)	
C35	0.66997 (10)	0.57242 (15)	0.45345 (8)	0.0426 (3)	
H35	0.6225 (13)	0.5946 (19)	0.3972 (12)	0.057 (4)*	
C36	0.66273 (9)	0.43910 (14)	0.50508 (8)	0.0370 (3)	
H36	0.6086 (12)	0.3684 (17)	0.4860 (10)	0.045 (4)*	

### Atomic displacement parameters (Å^2^)

**Table:**

	*U*^11^	*U*^22^	*U*^33^	*U*^12^	*U*^13^	*U*^23^
C1	0.0265 (5)	0.0272 (5)	0.0352 (5)	−0.0020 (4)	−0.0011 (4)	−0.0051 (4)
N11	0.0259 (4)	0.0261 (4)	0.0369 (5)	0.0014 (3)	0.0034 (3)	−0.0010 (3)
C12	0.0340 (5)	0.0403 (6)	0.0393 (6)	0.0027 (5)	0.0062 (4)	−0.0066 (5)
C13	0.0296 (5)	0.0384 (6)	0.0490 (7)	0.0004 (4)	0.0071 (5)	−0.0099 (5)
N14	0.0278 (5)	0.0288 (5)	0.0634 (6)	0.0031 (4)	0.0023 (4)	−0.0078 (4)
C15	0.0353 (6)	0.0338 (6)	0.0600 (7)	0.0080 (5)	0.0096 (5)	0.0089 (5)
C16	0.0298 (5)	0.0283 (5)	0.0591 (7)	0.0025 (4)	0.0092 (5)	0.0061 (5)
C21	0.0259 (5)	0.0220 (5)	0.0363 (5)	0.0016 (4)	−0.0006 (4)	0.0011 (4)
C22	0.0284 (5)	0.0273 (5)	0.0470 (6)	−0.0023 (4)	−0.0004 (4)	−0.0037 (4)
C23	0.0270 (5)	0.0311 (6)	0.0559 (7)	−0.0020 (4)	0.0051 (5)	0.0017 (5)
C24	0.0328 (5)	0.0287 (5)	0.0426 (6)	0.0060 (4)	0.0104 (4)	0.0056 (4)
F24	0.0458 (4)	0.0492 (4)	0.0562 (4)	0.0006 (3)	0.0237 (3)	−0.0013 (3)
C25	0.0343 (5)	0.0272 (5)	0.0357 (5)	0.0030 (4)	0.0007 (4)	0.0009 (4)
C26	0.0258 (5)	0.0267 (5)	0.0370 (5)	−0.0006 (4)	−0.0008 (4)	0.0010 (4)
C31	0.0291 (5)	0.0300 (5)	0.0312 (5)	0.0015 (4)	0.0028 (4)	−0.0034 (4)
C32	0.0333 (5)	0.0375 (6)	0.0393 (6)	−0.0047 (4)	−0.0035 (4)	0.0048 (5)
C33	0.0365 (6)	0.0373 (6)	0.0518 (7)	−0.0054 (5)	0.0045 (5)	0.0058 (5)
C34	0.0426 (6)	0.0368 (6)	0.0423 (6)	0.0095 (5)	0.0138 (5)	0.0110 (5)
F34	0.0578 (5)	0.0500 (5)	0.0649 (5)	0.0125 (4)	0.0191 (4)	0.0278 (4)
C35	0.0457 (6)	0.0478 (7)	0.0339 (6)	0.0133 (5)	−0.0014 (5)	0.0017 (5)
C36	0.0370 (6)	0.0373 (6)	0.0359 (5)	0.0028 (5)	−0.0041 (4)	−0.0074 (5)

### Geometric parameters (Å, º)

**Table:**

C1—N11	1.4808 (13)	C22—C23	1.3917 (16)
C1—C31	1.5211 (15)	C22—H22	0.989 (14)
C1—C21	1.5216 (14)	C23—C24	1.3684 (17)
C1—H1	0.996 (12)	C23—H23	0.978 (16)
N11—C12	1.4729 (14)	C24—F24	1.3601 (12)
N11—C16	1.4734 (14)	C24—C25	1.3828 (16)
C12—C13	1.5229 (16)	C25—C26	1.3849 (15)
C12—H12B	0.999 (14)	C25—H25	0.962 (14)
C12—H12A	0.996 (15)	C26—H26	0.970 (14)
C13—N14	1.4571 (16)	C31—C36	1.3901 (15)
C13—H13B	1.006 (15)	C31—C32	1.3920 (15)
C13—H13A	1.023 (14)	C32—C33	1.3909 (16)
N14—C15	1.4592 (16)	C32—H32	0.976 (14)
N14—H14	0.909 (15)	C33—C34	1.3706 (17)
C15—C16	1.5164 (16)	C33—H33	0.962 (17)
C15—H15B	1.039 (15)	C34—F34	1.3656 (13)
C15—H15A	1.015 (16)	C34—C35	1.3725 (19)
C16—H16B	0.976 (15)	C35—C36	1.3861 (18)
C16—H16A	0.993 (14)	C35—H35	0.963 (16)
C21—C22	1.3917 (14)	C36—H36	0.935 (15)
C21—C26	1.3928 (15)		
			
N11—C1—C31	113.31 (8)	C22—C21—C26	118.72 (10)
N11—C1—C21	110.63 (8)	C22—C21—C1	119.93 (9)
C31—C1—C21	109.48 (8)	C26—C21—C1	121.35 (9)
N11—C1—H1	109.0 (7)	C21—C22—C23	121.21 (10)
C31—C1—H1	106.2 (7)	C21—C22—H22	120.6 (8)
C21—C1—H1	108.0 (7)	C23—C22—H22	118.2 (8)
C12—N11—C16	108.32 (9)	C24—C23—C22	117.91 (10)
C12—N11—C1	110.38 (8)	C24—C23—H23	120.8 (9)
C16—N11—C1	109.22 (8)	C22—C23—H23	121.3 (9)
N11—C12—C13	110.93 (9)	F24—C24—C23	118.87 (10)
N11—C12—H12B	109.7 (8)	F24—C24—C25	118.05 (10)
C13—C12—H12B	109.3 (8)	C23—C24—C25	123.08 (10)
N11—C12—H12A	110.1 (8)	C24—C25—C26	118.07 (10)
C13—C12—H12A	108.0 (8)	C24—C25—H25	119.6 (8)
H12B—C12—H12A	108.9 (11)	C26—C25—H25	122.4 (8)
N14—C13—C12	109.73 (9)	C25—C26—C21	121.01 (9)
N14—C13—H13B	110.5 (8)	C25—C26—H26	119.6 (8)
C12—C13—H13B	108.2 (8)	C21—C26—H26	119.4 (8)
N14—C13—H13A	111.8 (8)	C36—C31—C32	118.08 (10)
C12—C13—H13A	108.9 (8)	C36—C31—C1	118.86 (9)
H13B—C13—H13A	107.6 (11)	C32—C31—C1	122.92 (9)
C13—N14—C15	108.94 (9)	C33—C32—C31	121.51 (10)
C13—N14—H14	111.7 (9)	C33—C32—H32	119.5 (9)
C15—N14—H14	110.4 (9)	C31—C32—H32	119.0 (9)
N14—C15—C16	109.28 (11)	C34—C33—C32	117.78 (11)
N14—C15—H15B	112.6 (8)	C34—C33—H33	119.0 (9)
C16—C15—H15B	108.4 (8)	C32—C33—H33	123.2 (9)
N14—C15—H15A	108.7 (9)	F34—C34—C33	118.49 (11)
C16—C15—H15A	108.9 (9)	F34—C34—C35	118.42 (11)
H15B—C15—H15A	108.8 (12)	C33—C34—C35	123.09 (11)
N11—C16—C15	111.17 (9)	C34—C35—C36	118.06 (11)
N11—C16—H16B	109.2 (9)	C34—C35—H35	119.3 (10)
C15—C16—H16B	109.5 (9)	C36—C35—H35	122.6 (10)
N11—C16—H16A	108.6 (8)	C35—C36—C31	121.46 (11)
C15—C16—H16A	109.3 (8)	C35—C36—H36	119.3 (9)
H16B—C16—H16A	109.0 (11)	C31—C36—H36	119.3 (9)
			
C31—C1—N11—C12	−62.03 (11)	C22—C23—C24—C25	−0.37 (17)
C21—C1—N11—C12	174.59 (8)	F24—C24—C25—C26	−179.60 (9)
C31—C1—N11—C16	178.99 (9)	C23—C24—C25—C26	0.45 (16)
C21—C1—N11—C16	55.61 (11)	C24—C25—C26—C21	−0.44 (15)
C16—N11—C12—C13	−56.84 (12)	C22—C21—C26—C25	0.36 (15)
C1—N11—C12—C13	−176.37 (9)	C1—C21—C26—C25	−179.73 (9)
N11—C12—C13—N14	59.56 (12)	N11—C1—C31—C36	145.48 (10)
C12—C13—N14—C15	−60.72 (12)	C21—C1—C31—C36	−90.50 (11)
C13—N14—C15—C16	60.98 (12)	N11—C1—C31—C32	−38.94 (14)
C12—N11—C16—C15	57.53 (13)	C21—C1—C31—C32	85.07 (12)
C1—N11—C16—C15	177.79 (10)	C36—C31—C32—C33	−1.06 (17)
N14—C15—C16—N11	−60.40 (13)	C1—C31—C32—C33	−176.67 (10)
N11—C1—C21—C22	−123.14 (10)	C31—C32—C33—C34	−0.02 (18)
C31—C1—C21—C22	111.29 (10)	C32—C33—C34—F34	−179.48 (10)
N11—C1—C21—C26	56.95 (12)	C32—C33—C34—C35	1.07 (19)
C31—C1—C21—C26	−68.62 (12)	F34—C34—C35—C36	179.60 (10)
C26—C21—C22—C23	−0.28 (16)	C33—C34—C35—C36	−0.95 (18)
C1—C21—C22—C23	179.81 (10)	C34—C35—C36—C31	−0.22 (17)
C21—C22—C23—C24	0.28 (17)	C32—C31—C36—C35	1.19 (17)
C22—C23—C24—F24	179.68 (10)	C1—C31—C36—C35	176.98 (10)

### Hydrogen-bond geometry (Å, º)

**Table:**

*D*—H···*A*	*D*—H	H···*A*	*D*···*A*	*D*—H···*A*
C25—H25···F24^i^	0.962 (14)	2.424 (14)	3.2720 (13)	146.8 (10)
C25—H25···F34^ii^	0.962 (14)	2.533 (14)	3.1998 (13)	126.5 (10)

Symmetry codes: (i) −*x*+1, −*y*+1, −*z*+2; (ii) *x*, −*y*+3/2, *z*+1/2.
